# Flower-Shaped PCR Scaffold-Based Lateral Flow Bioassay for *Bacillus cereus* Endospores Detection

**DOI:** 10.3390/ijms252011286

**Published:** 2024-10-20

**Authors:** Jingjing Tian, Zhuyi Zhang, Yaning Shi, Zichao Wu, Yuting Shao, Limin Wang, Xinglian Xu, Zhihong Xin

**Affiliations:** 1 Key Laboratory of Food Processing and Quality Control, State Key Lab of Meat Quality Control and Cultured Meat Development, College of Food Science and Technology, Nanjing Agricultural University, Nanjing 210095, China; tianjingjing@njau.edu.cn (J.T.); zyzhang_0615@163.com (Z.Z.); yanings@njau.edu.cn (Y.S.); zichaowu_jason@163.com (Z.W.); 2018208019@njau.edu.cn (Y.S.); xlxu@njau.edu.cn (X.X.); 2 College of Plant Protection, Nanjing Agricultural University, Nanjing 210095, China; wlm@njau.edu.cn

**Keywords:** flower-shaped PCR scaffold, test strip, *Bacillus cereus* endospores, lateral flow biosensor, point-of-care testing (POCT)

## Abstract

*Bacillus cereus*, a foodborne pathogen, produces resilient endospores that are challenging to detect with conventional methods. This study presents a novel Flower-Shaped PCR Scaffold-based Lateral Flow Biosensor (FSPCRS-LFB), which employs an aptamer-integrated PCR scaffold as capture probes, replacing the traditional streptavidin-biotin (SA-Bio) approach. The FSPCRS-LFB demonstrates high sensitivity and cost-efficiency in detecting *B. cereus* endospores, with a limit of detection (LOD) of 4.57 endospores/mL a visual LOD of 10^2^ endospores/mL, and a LOD of 6.78 CFU/mL for endospore-cell mixtures. In chicken and tea samples, the platform achieved LODs of 74.5 and 52.8 endospores/mL, respectively, with recovery rates of 82.19% to 97.88%. Compared to existing methods, the FSPCRS-LFB offers a 3.7-fold increase in sensitivity while reducing costs by 26% over the SA-Bio strategy and 87.5% over rolling circle amplification (RCA). This biosensor provides a rapid, sensitive and cost-effective solution for point-of-care testing (POCT) of *B. cereus* endospores, expanding detection capabilities and offering novel approaches for pathogen detection.

## 1. Introduction

*Bacillus cereus* is a ubiquitous Gram-positive foodborne pathogen that frequently contaminates high-protein foods such as meat, dairy products, and vegetables [1]. This bacterium produces resilient endospores and various toxins [2], including enterotoxins that can enter the human body through endospores and, combined with other toxic factors, cause gastrointestinal infections and symptoms like diarrhea [3,4]. *B. cereus* endospores, typically 0.8–1.2 µm in diameter, are highly resistant, dormant structures formed under extreme environmental conditions [4]. Their low metabolic activity and well-organized outer structure allow them to survive in adverse conditions, such as high temperatures, radiation, ultraviolet light, desiccation, and chemical exposure [5]. These characteristics enable rapid bacterial revival, and resumption of normal functions once favourable conditions return [6]. Heat treatments, although effective in eliminating other microorganisms, may inadvertently promote *B. cereus* endospore growth by reducing microbial competition [7,8]. The hydrophobic nature of the endospores further enhances pathogenicity by facilitating adhesion to epithelial cells, increasing the risk of infection [9].

Current methods for detecting *B. cereus* primarily include traditional plate culture techniques, molecular biology methods [10,11], and sensor-based technologies [12,13]. For endospore detection, molecular techniques like PCR (polymerase chain reaction) [14] are commonly used alongside traditional culture methods. Fisher developed a PCR approach utilizing magnetic beads and aptamers, where aptamers on magnetic beads capture endospores, and magnetic enrichment enables detection at a limit of 10^3^ CFU/mL [15]. Additionally, dipicolinic acid (DPA), which constitutes 5–15% of the endospore’s dry weight, is an indirect marker for quantifying endospore content [16]. Gültekin et al. used gold-silver nanoclusters with a molecularly imprinted polymer (MIP) nanoshell, using DPA as a template, to detect DPA through chelation [17]. MIPs are highly selective materials prepared through molecular imprinting technology, mimicking the recognition ability of antibodies or enzymes. Due to their high stability, low cost, and ease of preparation, MIPs are widely used in antibiotic detection, particularly in solid-phase extraction and sensors, where they enhance the sensitivity and selectivity of detection [18]. Lateral flow immunoassays are also widely employed. For instance, Wang et al. used magnetic beads modified with endospore-specific antibodies to capture endospores [19]. Despite their sensitivity and specificity, these methods often face challenges such as being time-consuming, expensive, and requiring specialized expertise [20,21,22], hindering timely identification and management of endospore contamination in food safety. This highlights the need for novel detection methods that overcome these limitations.

Lateral flow assay (LFA), also known as lateral flow immunoassay or biosensor, is a promising point-of-care testing tool renowned for its rapid detection, high selectivity, low cost, ease of production, stability, and biodegradability [23,24]. First introduced in the 1960s for detecting serum proteins and human chorionic gonadotropin (hCG), LFA has since been applied to a wide range of targets, including proteins, nucleic acids, small molecules, pathogens, and cancer cells [25]. A typical LFA consists of a sample pad, conjugate pad, nitrocellulose membrane (NC membrane), absorbent pad, and backing card, with samples moving through these pads via capillary action [26]. There are two main types of LFA: the sandwich assay [27] and the competitive assay [28]. In the sandwich assay, the target analyte binds to the signal probe and is captured by immobilized antibodies at the Test-line (T-line), forming a visible band, while unbound probes move to the Control-line (C-line), confirming test validity [29]. In the competitive assay, the target competes with the signal probe for binding at the T-line. If the target is present, the T-line remains unmarked, while unbound probes are captured at the C-line, ensuring test accuracy [30]. Common capture probes include antibodies and aptamers [31,32]. Aptamers, short single-stranded oligonucleotides selected via Systematic Evolution of Ligands by Exponential Enrichment (SELEX) [33,34], offer advantages over antibodies, such as flexible binding, shorter selection time, broader target range, and low immunogenicity [35]. For example, Ying et al. developed an LFA using aptamer A3P and magnetic nanoparticles (MNPs) to detect *Vibrio parahaemolyticus* with a detection limit of 2.6 × 10^3^ cells [36]. Similarly, Song et al. created a visual biosensor incorporating dual aptamers and rolling circle amplification (RCA) to detect *V. parahaemolyticus*, achieving a detection limit of 10 CFU/mL [37].

When designing a nucleic acid-based lateral flow biosensor (LFB), a key consideration is the stable immobilization of nucleic acids on the NC membrane while maintaining their functional integrity [31]. Standard immobilization methods include covalent and non-covalent bonding [38]. Covalent methods, such as aldehyde-amine coupling, provide better stability but require complex chemical modifications [39]. In contrast, non-covalent approaches, like hydrogen bonding and hydrophobic interactions, are simpler but less suitable for aptamers due to their tendency to desorb during chromatography [40]. To overcome these limitations, researchers developed a streptavidin-biotin-based (SA-Bio) immobilization strategy, exploiting the strong affinity between streptavidin and biotin [41]. Additionally, the cost of streptavidin-biotin reagents limits widespread use [31]. An alternative approach is RCA, which generates long-chain DNA molecules that stably adhere to the NC membrane [42]. RCA products form flower-shaped scaffolds with increased surface area and enhanced nuclease resistance, making them particularly useful for LFA development [43,44]. While RCA produces single-stranded DNA, PCR generates double-stranded DNA, offering greater stability, easier quantification, and more consistent amplification. These advantages make PCR more suitable for many LFA applications, especially where long-term stability and reproducibility are essential [45,46]. In 2022, our research group applied the traditional molecular biology technique, PCR, to the preparation of micro- and nanoscale nanomaterials [47].

To overcome the challenges mentioned above, this study introduces a novel flower-shaped PCR scaffold strategy for immobilizing capture probes on a LFB. Spacer 18 linkers, composed of a hexaglycol chain with 18 atoms (12 carbons and 6 oxygens), were incorporated into the primers. Known for its hydrophobicity, Spacer 18 introduces long spacer arms into oligonucleotides and inhibits polymerase activity, preventing further chain elongation [48]. In this study, the *B. cereus*-specific aptamer was linked to the 5′ end of the primers using Spacer 18, generating a modified primer pair. During PCR amplification, Spacer 18 halts chain extension, leaving the aptamer segment single-stranded. The resulting double-stranded DNA (dsDNA) contains *B. cereus*-specific aptamers at both ends. The dsDNA is then incubated with Mg^2+^ and pyrophosphate (PPi), which forms magnesium pyrophosphate upon binding to excess Mg^2+^. Once the solubility limit is reached, nucleation and growth occur, with dsDNA depositing onto the magnesium pyrophosphate surface, ultimately forming a flower-shaped PCR scaffold [47]. This scaffold can be tailored to capture various targets by modifying the single-stranded DNA (ssDNA) sequence. The T-PCR and C-PCR scaffolds are immobilized as capture probes on the T-line and C-line. Due to its target recognition and complementarity to the signal probe, the T-PCR scaffold captures both *B. cereus* cells and endospores, as well as signal probes. The C-PCR scaffold, through its complementarity to the signal probe, captures the signal probe at the C-line, ensuring the accuracy of the biosensor. A polyA-cDNA sequence was designed as a signal probe, consisting of a polyadenine (polyA) anchor, a fragment complementary to the C-line (cDNA_C_), and a fragment complementary to the T-line (cDNA_T_). This sequence was conjugated to gold nanoparticles (AuNPs) using a low-pH method, with bovine serum albumin (BSA) added as a blocking agent to minimize false negatives and enhance sensitivity. In summary, this study developed a flower-shaped PCR scaffold-based lateral flow biosensor (FSPCRS-LFB) utilizing aptamer-integrated PCR scaffolds as capture probes and AuNPs@polyA-cDNA nanoprobes as signal probes for the rapid detection of *B. cereus* endospores and endospore-cell mixtures. The biosensor demonstrated efficient, sensitive, and cost-effective point-of-care testing (POCT), addressing the limitations of traditional methods, such as long processing times, high costs, and the need for specialized personnel. Additionally, it expands the sensor’s detection capabilities, providing a novel approach for detecting foodborne pathogens. The structure of the FSPCRS-LFB sensor developed in this study is shown. The sensor consists of, from left to right, a sample pad, a conjugate pad, an NC membrane, and an absorbent pad. T and C lines are fixed on the NC membrane, while the signal probe AuNPs@polyA-cDNA is immobilized on the conjugate pad. The T line contains a T-PCR scaffold to capture B. cereus spores and the signal probes, while the C line contains a C-PCR scaffold to capture the signal probes.

The article presents a universal lateral flow assay paper-based sensor platform based on a competitive method. In principle, universal detection can be achieved by simply modifying the aptamer sequence specific to the target. However, the detection mechanism relies on both the aptamer’s recognition of the target and the complementary hybridization of nucleic acid sequences. Balancing these interactions is crucial—if the aptamer’s binding affinity is weaker than the nucleic acid hybridization, false negatives may occur, while stronger hybridization could result in blurred bands, making visual detection difficult. Thus, it is essential to evaluate binding strengths and adjust nucleic acid sequences accordingly during sensor design. Due to the programmability of nucleic acid sequences, we can design various sequences to detect common pathogenic microorganisms such as *Escherichia coli* and *Staphylococcus aureus*. This approach could make the nucleic acid lateral flow bioassay an efficient, sensitive, simple, and rapid tool for detecting a wide range of pathogens.

## 2. Result and Discussions

### 2.1. Principle of the Flower-Shaped PCR Scaffold-Based Lateral Flow Biosensor

**Figure 1:**
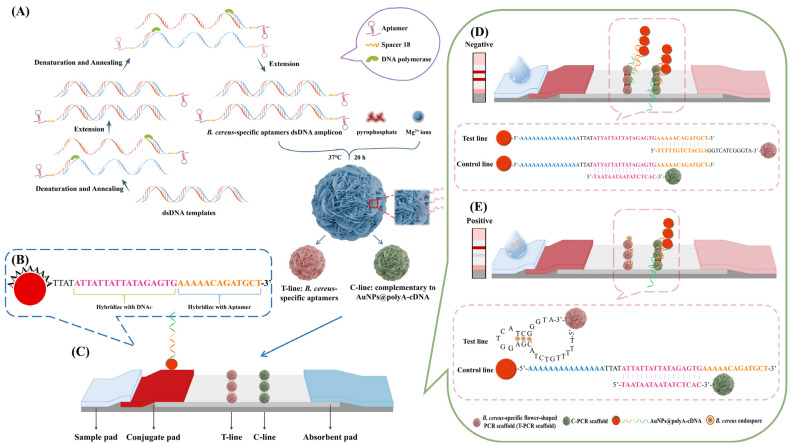
Design principle of a flower-shaped PCR scaffold-based lateral flow biosensor platform. (**A**) The preparation process of a flower-shaped PCR scaffold was performed by modifying the aptamer sequences. The T-PCR scaffold and C-PCR scaffold were obtained, respectively. (**B**) The structure of AuNPs@polyA-cDNA contains a polyA segment as an anchoring block, a cDNAa segment complementary to the T-PCR scaffold, and a cDNAc segment complementary to the C-PCR scaffold. (**C**) Structure of the test strip. (**D**) Negative test: in the absence of *B. cereus* endospores, the T and C lines display red bands. (**E**) Positive test: Only the C line displays a red band in the presence of *B. cereus* endospores.

The preparation process of the flower-shaped PCR scaffold is shown in Figure 1A. Aptamer sequences are introduced at both ends of the primers using Spacer 18, which stops DNA single-strand elongation to double-stranded ones before its position. This creates an amplicon with single-stranded aptamer sequences at both ends, serving as capture probes, while the central double-stranded region supports self-assembly, forming the aptamer-rich flower-shaped PCR scaffold.

The AuNPs@polyA-cDNA probe consists of three components: polyA, cDNAc, and cDNAa (Figure 1B). The polyA segment, containing 15 adenine bases, anchors to the AuNPs surface via electrostatic adsorption. As shown in Figure 1C, this paper-based sensor utilizes competitive binding between *B. cereus* endospores and AuNPs@polyA-cDNA at the T line. The cDNAc fragment is complementary to the microflower sequence immobilized at the C line, while cDNAa is complementary to the aptamer microflower sequence at the T line, enabling binding at both T and C lines.

In the absence of *B. cereus* endospores, the cDNAa fragment of AuNPs@polyA-cDNA hybridizes with the aptamer at the T line, generating a distinct signal from AuNP accumulation (Figure 1D). In contrast, when *B. cereus* endospores are present, their binding to the aptamer inhibits the hybridization of AuNPs@polyA-cDNA with the aptamer, weakening the signal at the T line (Figure 1E). The C-PCR scaffold captures excess AuNPs@polyA-cDNA by hybridizing with the cDNAc fragment. Thus, regardless of the presence of *B. cereus* endospores, the probe is captured at the C line, producing a red band.

### 2.2. Characterization of Flower-Shaped PCR Scaffolds

**Figure 2:**
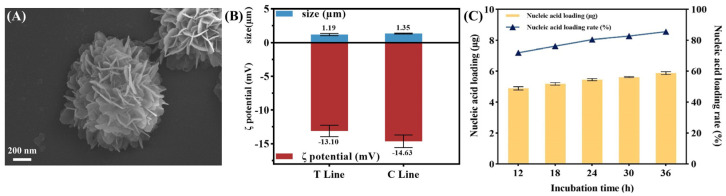
Preparation and characterization of flower-shaped PCR scaffolds. Scanning electron microscopy (SEM) (**A**) revealed the loose, porous and multilayered flower-like morphology of the PCR scaffold. Dynamic Light Scattering (DLS) (**B**) and ζ-potential analysis characterized the average particle size (1.3 µm) and the electro-negativity of the PCR scaffold, respectively. (**C**) The dsDNA loading efficiency of flower-shaped PCR scaffolds was confirmed by (initial amount of nucleic acid added − remaining nucleic acid in the supernatant)/initial amount of nucleic acid added, with a capacity exceeding 70%, indicating a substantial presence of PCR amplicons.

The flower-shaped PCR scaffold is a highly stable nano- to microscale structure formed by DNA self-assembly on a magnesium pyrophosphate backbone (Figure 2A). By modifying the nucleic acid sequences at the primer ends, we constructed two distinct flower-shaped PCR scaffolds: the T-PCR scaffold, which captures the target and is complementary to the AuNPs@polyA-cDNA nanoprobe, and the C-PCR scaffold, serving as a complementary sequence to the AuNPs@polyA-cDNA nanoprobe. The prepared scaffold has a distinct flower-like structure with a particle size of approximately 1.2–1.4 µm (Figure 2B), ensuring stable deposition on the NC membrane without migration due to capillary action. To assess DNA loading capacity, we measured nucleic acid concentration in the supernatant after the first centrifugation and calculated loading efficiency as: loading efficiency = (initial nucleic acid − remaining nucleic acid)/initial nucleic acid. As shown in Figure 2C, the scaffold achieved over 70% loading efficiency, indicating a high capacity for target sequence binding, which enhances its ability to recognize *B. cereus* endospores and cells, significantly improving sensor sensitivity.

**Figure 3:**
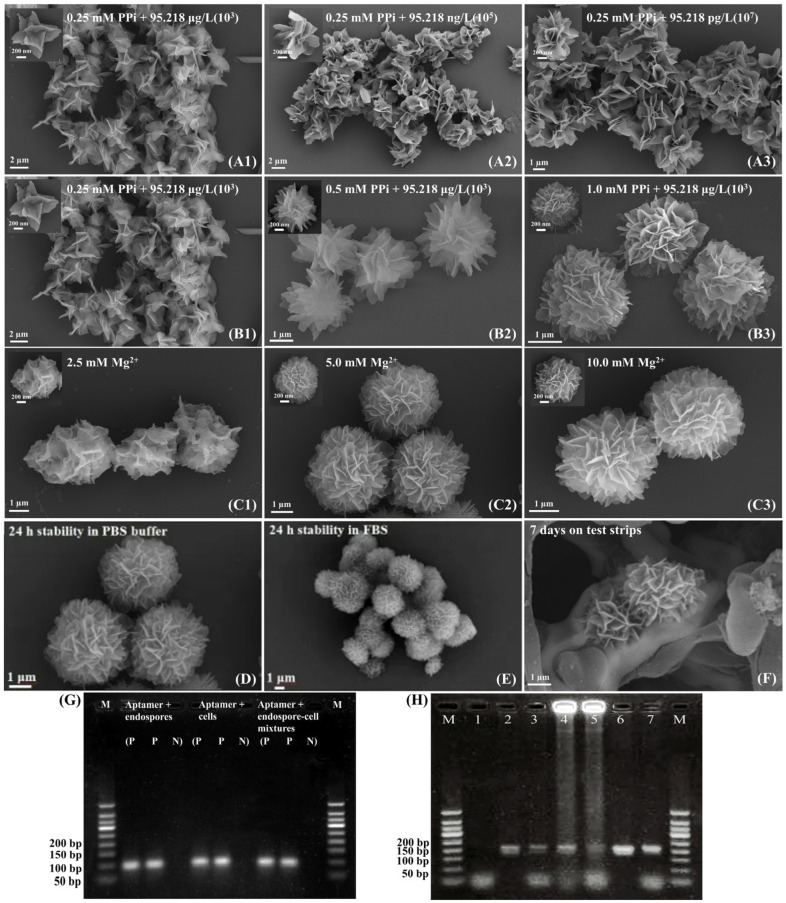
Morphological tunability, stability and endospores captive property of Flower-Shaped PCR scaffolds. Lower nucleic acid concentrations at a constant pyrophosphate level result in denser flower-like folds (**A1**–**A3**). Lower pyrophosphate concentrations at a constant nucleic acid level produce sparser folds (**B1**–**B3**). Magnesium ion concentration is critical for maintaining the flower-like morphology of PCR scaffolds under consistent nucleic acid and pyrophosphate conditions (**C1**–**C3**). The morphology of flower-like PCR scaffolds by SEM characterization after one week at 37 °C in PBS buffer (**D**), 100% fetal bovine serum (**E**), and on test strips as capture probes (**F**). Agarose gel electrophoresis (AGE) characterized the capture abilities of nucleic acid aptamers for *B. cereus* endospores, bacterial cells and endospore-cell mixtures (**G**), as well as the capture efficiency of flower-shaped PCR scaffolds for endospores (**H**): polyA-cDNA, T-PCR scaffold, T-PCR scaffold + polyA-cDNA, endospores + T-PCR scaffold, endospores + T-PCR scaffold + polyA-cDNA, C-PCR scaffold, C-PCR scaffold + polyA-cDNA.

To investigate factors influencing the scaffold’s morphology, we adjusted DNA, PPi, and Mg^2+^ concentrations during preparation and characterized the structures using SEM (Figure 3A–C). As seen in Figure 3(A1–A3), with a fixed Mg_2_P_2_O_7_ concentration of 0.25 mM, decreasing DNA concentration led to denser folds and a more distinct flower-like structure. Figure 3(B1–B3) shows that lower pyrophosphate concentrations resulted in sparser folding, while Figure 3(C1–C3) highlights the critical role of Mg^2+^ in maintaining flower-like morphology under fixed DNA and PPi conditions. These results demonstrate that scaffold morphology can be controlled by adjusting DNA, PPi, and Mg^2+^ concentrations.

As a capture probe for LFA, the scaffold must be stable. To assess this, we stored the scaffold in PBS buffer and fetal bovine serum (FBS) for 24 h and fixed it on an NC membrane for 7 days at 37 °C. SEM analysis confirmed stable morphology in both PBS and FBS (Figure 3D,E). Using the Arrhenius equation (k = Aexp (−Ea/RT)), we found that storage at 37 °C for 7 days is equivalent to preservation at 4 °C for one year (Figure 3F), confirming its stability as an LFA capture probe. To verify the aptamer’s capture ability, we incubated it with spores, cells, and endospore-cell mixtures. After centrifugation, the target-aptamer complex was collected, and the aptamer was released by heat denaturation. The supernatant was then used for PCR verification. As shown in Figure 3G, “P” represents the positive group (spores, cells, and endospore-cell mixtures), and “N” represents the negative control (use *Escherichia coli*, *Staphylococcus aureus*, and *Salmonella* as targets). The results show that the aptamer specifically captures *B. cereus* spores and cells, but not other bacteria like *E. coli*. The recognition and capture ability of the flower-shaped PCR scaffolds were further tested. Flower-shaped PCR scaffolds, polyA-cDNA, and spores were incubated, followed by agarose gel electrophoresis. In Figure 3H, lanes 1–7 show polyA-cDNA, T-PCR scaffold, T-PCR scaffold + polyA-cDNA, spores + T-PCR scaffold, spores + T-PCR scaffold + polyA-cDNA, C-PCR scaffold, and C-PCR scaffold + polyA-cDNA, respectively. Lane 3 shifts slightly compared to Lane 2 due to 13nt hybridization, and Lane 7 shifts up compared to Lane 6 due to 17nt hybridization. The large spores were captured by the T-PCR scaffold, resulting in bright bands in lanes 4 and 5. These results confirm that the flower-shaped PCR scaffold retains target recognition and hybridization capabilities, making it a promising LFA capture probe.

### 2.3. Characterization of AuNPs@polyA-cDNA Nanoprobes

**Figure 4:**
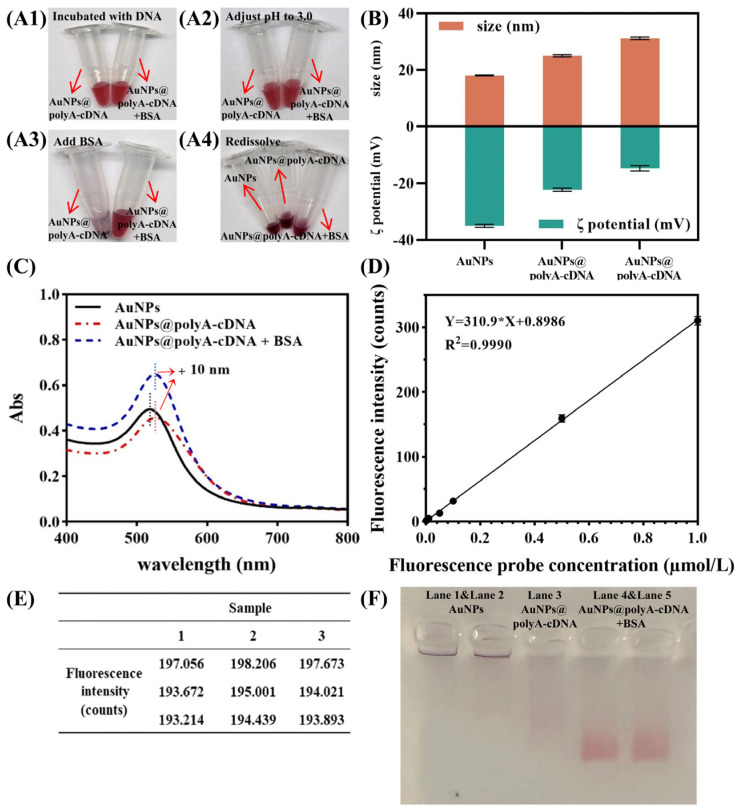
Preparation and characterization of AuNPs@polyA-cDNA signal probe. (**A1**–**A4**) The image depicts the state changes of AuNPs@polyA-cDNA signal probes during preparation, confirming their strong color signal and monodispersity. (**B**) AGE tests assess the dispersion and migration properties of the signal probes. (**C**) DLS and ζ-potential measurements indicate that, following nucleic acid modification and BSA blocking, the signal probes have an average particle size of 31.2 nm and a ζ-potential of −14.7 mV. (**D**) A 10 nm red shift in the UV-Vis absorption spectrum confirms the successful modification of AuNPs with polyA-cDNA probes and BSA as a blocking agent. (**E**,**F**) Quantification using AF488-polyA-cDNA shows a nucleic acid adsorption efficiency of 73.5%, based on the standard curve (**E**) and observed fluorescence intensity (**F**).

AuNPs are widely used as signal molecules in sensors due to their ease of preparation, high sensitivity, low cost, and modifiability [49,50,51,52]. However, conventional thiol-based Au-S conjugation is costly and time-consuming. In this study, we introduced a polyadenine (polyA) sequence to simplify and reduce the cost of nucleic acid-AuNP conjugation using a low-pH method. Under acidic conditions (pH < 4), protonated adenine binds to negatively charged AuNPs via electrostatic interactions. Characterization confirmed successful conjugation: Figure 4(A1–A4) illustrates the preparation process of AuNPs@polyA-cDNA, during which the solution consistently remained a bright cherry red. The addition of nucleic acids and BSA did not disrupt the original structure of the AuNPs. Particle size increased by ~10 nm, and zeta potential shifted by ~10 mV (Figure 4B). A ~10 nm red shift in maximum absorption (Figure 4C) further validated the conjugation. Fluorescence measurements using AF488-labeled polyA-cDNA showed an adsorption rate of 73.5% (Figure 4D,E), indicating efficient nucleic acid loading and enhanced sensor sensitivity.

However, AuNPs@polyA-cDNA exhibited nonspecific adsorption and poor migration on test strips, likely due to incomplete nucleic acid surface coverage and the high surface energy of AuNPs. To address this, bovine serum albumin (BSA) was introduced as a blocking agent. At pH < 4.7, BSA becomes positively charged, adsorbing onto AuNPs without displacing nucleic acids. Optimization results showed improved performance: particle size increased by 6 nm, zeta potential shifted by ~10 mV (Figure 4B), and gel electrophoresis revealed enhanced migration and compact structure for AuNPs@polyA-cDNA with BSA (Figure 4F). These findings demonstrate that BSA effectively enhances the stability and migration of AuNPs@polyA-cDNA, supporting its application in sensor systems.

### 2.4. Analytical Performance of FSPCRS-LFB for Endospores Detection

**Figure 5:**
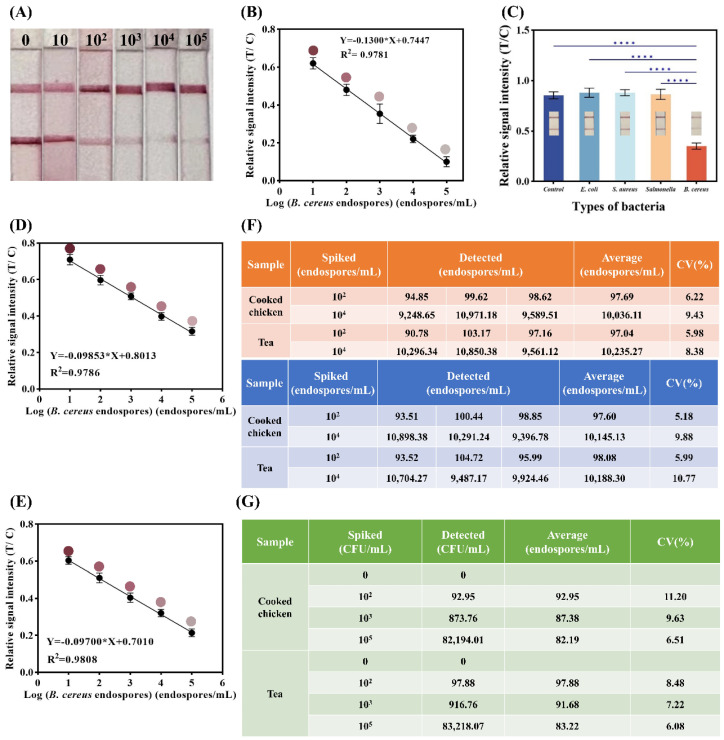
Analytical performance of the flower-shaped PCR scaffold-based lateral flow biosensor for endospores detection. (**A**) Detection results for various endospores concentrations (0, 10, 10^2^, 10^3^, 10^4^, 10^5^ endospores/mL). Endospores are visually detectable at concentrations of 10^2^ endospores/mL. (**B**) Linear correlation between the logarithm of endospores concentration and T/C signal intensity within the range of 10 to 10⁵endospores/mL; LOD calculated as 4.57 endospores/mL. (**C**) Specificity of FSPCRS-LFB against *E. coli*, *S. aureus*, and *Salmonella*, showing high specificity for *B. cereus* endospores. **** (*p* < 0.0001). (**D**,**E**) Linear correlation between the logarithm of endospores concentration and T/C signal intensity in chicken (**D**) and tea infusion (**E**), with LODs of 74.5 endospores/mL and 52.8 endospores/mL, respectively. (**F**) Reproducibility of FSPCRS-LFB for *B. cereus* endospores in actual samples, with intra- and inter-assay variations below 12%. (**G**) Spiked recovery rates of *B. cereus* endospores, ranging from 82.19% to 97.88%, with CVs below 12%.

Under optimized conditions, various concentrations of *B. cereus* endospores were prepared to evaluate the performance of the flower-shaped PCR scaffold-based lateral flow biosensor. As shown in Figure 5A, increasing endospore concentrations gradually faded the T-line color and decreased signal strength. Endospore concentrations of 10^2^ endospores/mL were easily distinguishable by the naked eye. The T/C signal ratio, measured using a chromatographic reader, showed a linear relationship with spore concentration of 10 to 10^5^ endospores/mL (Figure 5B). The detection limit was calculated as 4.57 endospores/mL using the formula 3σ/k (σ = standard deviation) [28]. These findings demonstrate the assay’s high sensitivity, enabling rapid detection of *B. cereus* endospores within 15 min.

To assess specificity, the sensor was tested against other common foodborne bacteria, including *E. coli*, *S. aureus*, *Salmonella*, along with a blank control. *B. cereus* endospores were tested at 10^2^ endospores/mL, while other bacteria were tested at 10^5^ CFU/mL. As shown in Figure 5C, only the *B. cereus* group showed a significant reduction in signal intensity, confirming the sensor’s high selectivity for *B. cereus* endospores and validating the aptamer’s specificity.

For real-sample evaluation, varying concentrations of endospores were added to chicken and tea broth samples. Detection limits were 74.5 and 52.8 endospores/mL, respectively, with a linear range of 10 to 10^5^ endospores/mL (Figure 5D,E). Reproducibility was assessed using three batches of test strips and reagents to detect endospore concentrations of 10^2^ and 10^4^ endospores/mL in blank samples. As shown in Figure 5F, intra- and inter-assay variations were below 12%, indicating good reproducibility. Spiking recovery experiments with different endospore concentrations showed recovery rates between 82.19% and 97.88%, with a coefficient of variation (CV) below 12% (Figure 5G).

### 2.5. Analytical Performance for Endospore-Cell Mixtures Detection

**Figure 6:**
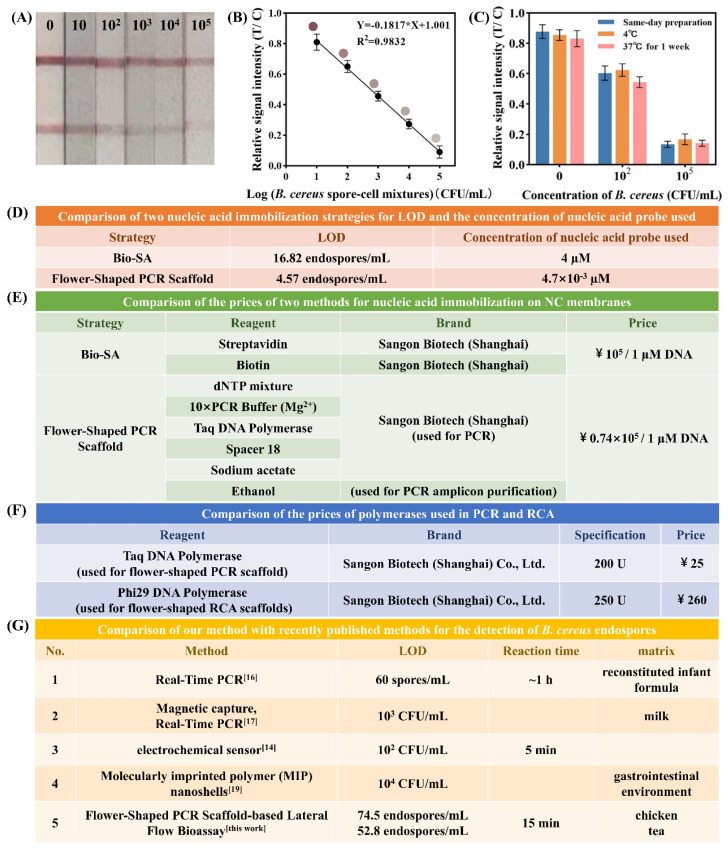
Analysis of endospore-cell mixtures. (**A**) Detection results for endospore-cell mixtures at concentrations of 0, 10, 10^2^, 10^3^, 10^4^, and 10^5^ CFU/mL. Mixtures are visually detectable at 10^3^ CFU/mL. (**B**) Linear correlation between the logarithm of endospore-cell mixtures concentration and T/C signal intensity from 10 to 10⁵ CFU/mL; LOD is 6.78 CFU/mL (Each colored circle represents the band color at the corresponding concentration). (**C**) Stability of FSPCRS-LFB test strips stored at 37 °C for one week, equivalent to one year at 4 °C, showing consistent T/C signal intensities across all concentrations (0, 10^2^, 10^5^ CFU/mL), indicating excellent stability for long-term storage. (**D**) Comparison of two nucleic acid immobilization strategies for LOD and the concentration of nucleic acid probe used. (**E**) Comparison of the prices of two methods for nucleic acid immobilization on NC membranes. (**F**) Comparison of the prices of polymerases used in PCR and RCA. (**G**) Comparison of our method with recently published methods for detecting *B. cereus* endospores.

To evaluate the sensor’s performance across different concentrations of endospore-cell mixtures, various concentrations were prepared. As shown in Figure 6A,B, signal intensity decreased with increasing mixture concentration. The visual detection limit was 10^2^ CFU/mL, with a calculated detection limit of 6.78 CFU/mL, and a linear detection range of 10 to 10^5^ CFU/mL.

To assess sensor stability, test strips were stored at 4 °C and 37 °C for 7 days, with freshly prepared strips as controls. Samples containing 0, 10^2^, and 10^5^ CFU/mL of endospore-cell mixtures were tested. Figure 6C shows no significant differences in signal intensity across storage conditions. Based on the Arrhenius equation, which equates 7 days at 37 °C to 1 year at 4 °C, the strips can be stored at 4 °C for at least one year.

Compared to SA-Bio, the flower-shaped PCR scaffold-based biosensor demonstrated a 3.7-fold increase in sensitivity and a 26% reduction in cost (Figure 6D,E). Additionally, its cost is only 12.5% of that for RCA (Figure 6F). In summary, the FSPCRS-LFB offers higher sensitivity and faster detection than recent *B. cereus* endospore detection methods (Figure 6G).

## 3. Materials and Methods

### 3.1. Reagents, Consumables and Apparatus

Reagents: The 20-mer *B. cereus*-binding aptamer (5′-ATGGGCTACTGGAGCATCTG-3′) was adopted from the literature [53]. All nucleic acid sequences (Tables S1 and S2) were purchased from Sangon Biotech Co., Ltd. (Shanghai, China). The PCR reagents used for the flower-shaped PCR scaffold were purchased from TaKaRa and TIANGEN Biotech (Beijing) Co., Ltd. (Beijing, China). K_4_P_2_O_7_ and MgCl_2_ were purchased from Macklin (Shanghai, China). HAuCl_4_ and trisodium citrate used for AuNPs@polyA-cDNA were purchased from Macklin (Shanghai, China), and BSA was purchased from Solarbio (Beijing, China). All reagents were of analytical grade and used as received. All solutions were prepared with ultrapure water (18.2 MΩ/cm) from a Millipore Milli-Q water purification system (Billerica, MA, USA).

Consumables: The absorbent pad (SX27), sample pad (KB50), conjugate pad (SB08), nitrocellulose (NC) membrane (Sartorius CN140), and PVC plastic adhesive backing (SM31–40) used in the experiments were purchased from Shanghai Goldbio Biotechnology Co., Ltd. (Shanghai, China).

Apparatus: The film applicator was purchased from Shanghai Jiening Biotechnology Co., Ltd. (Shanghai, China). Each lateral flow biosensor strip was cut using a Deli paper cutter, and the signals of the test strip were scanned by a chromatography reader.

### 3.2. Preparation and Characterization of Flower-Shaped PCR Scaffold

To fabricate a flower-shaped PCR scaffold, a 50 μL PCR system was prepared with templates, 0.025 U·μL^−1^ rTaq DNA polymerase, 0.4 μM each of forward and reverse primers, 250 μM dNTPs, and 1× PCR buffer (Mg^2+^ plus). The PCR program included 95 °C for 5 min, followed by 40 cycles of 95 °C for 60 s, 56 °C for 60 s, and 72 °C for 60 s, with a final extension at 72 °C for 10 min. The PCR products were purified and recovered. For one-pot flower-shaped PCR scaffold assembly, a 200 μL system containing various PCR amplicon concentrations, 2.0 mM PPi, 8.5 mM Mg^2+^, and 1× PCR buffer was incubated at 37 °C for 20 h. After centrifugation at 12,000 rpm for 20 min, the precipitate was washed with RNase-free water, dried at 37 °C, and stored at 4 °C. Samples were then deposited onto silicon wafer, coated with platinum, and observed using SEM.

### 3.3. Preparation and Optimization of AuNPs@polyA-cDNA

Gold nanoparticles (AuNPs) with an average diameter of 20 nm were synthesized using the sodium citrate reduction method [54]. In brief, 93 mL of water and 1 mL of 1% HAuCl_4_ were heated in a 250 mL flask for 1–2 min, followed by adding 3 mL of freshly prepared 1% trisodium citrate. The solution was heated with stirring until the color changed from pale yellow to wine red. After 10 min of heating, the solution was cooled in a water bath and stored at 4 °C.

The AuNPs@polyA-cDNA probe was prepared by mixing 1–3 µL of polyA-cDNA (100 µM) with 200 µL of AuNPs (10 nM), incubating at room temperature for 5 min, then adding 500 mM citrate buffer (pH 3.0, final concentration is 10 nM) and incubating for 1 h. After adjusting the pH with a HEPES buffer (pH 7.6, three times the volume of the previous buffer), the mixture was incubated for an additional 2 h, centrifuged, and resuspended in HEPES buffer (pH 7.4, containing 0.25% Tween-20) [55].

Optimization step: For BSA addition, 20 mg/mL BSA in citrate buffer was added after the 1-h incubation, followed by 2 h of further incubation. The final probe was stored at 4 °C [56].

### 3.4. Fabrication of the Flower-Shaped PCR Scaffold-Based Lateral Flow Biosensor

The sensor comprises a sample pad, conjugate pad, NC membrane, absorbent pad, and PVC backing card. The sample pad and conjugate pad were treated for 30 min with different solutions: 0.05 M Tris-HCl (pH 8.0) containing 0.25% Triton X-100, 0.15 M NaCl, and 1% BSA for the sample pad, and 0.01 M PBS containing 1% sucrose, 1% BSA, and 0.2% Tween-20 for the conjugate pad, respectively. Pre-prepared AuNPs@polyA-cDNA was sprayed onto the conjugate pad and dried at 37 °C for 2 h. The T-PCR scaffold (T-line) and C-PCR scaffold (C-line) were resuspended in 4× SSC buffer and sprayed onto the NC membrane, spaced 5 mm apart, then dried at 37 °C for 1 h. The components were assembled, and the test strips were cut to 4 mm widths and stored in sealed bags.

In the analysis procedure, *B. cereus* endospores or endospore-cell mixtures were diluted to various concentrations using running buffer (1× BB, 0.25% Tween-20) and applied to the sample pad of the test strip. The solution migrated upward via capillary action. After 15 min, when the band color stabilized, the strips were photographed, and the T and C-line signal intensities were measured using a chromatography reader. The T/C signal ratio was recorded for further analysis.

### 3.5. Sample Assay Procedures

To assess the sensor’s practical application, it was used to detect *B. cereus* endospores in chicken and tea broth samples. A 1 g chicken sample was vortexed in 1 mL of 3% trichloroacetic acid solution for 5 min and centrifuged at 10,000 rpm for 10 min. The supernatant was neutralized with 1 M NaOH and diluted 20 times. For the tea sample, 1 g of dried tea was soaked in a mixture of 20 mL ethanol and 25 mL ultrapure water for 30 min, then filtered. Finally, 0.5 mL of each chicken, tea broth, and endospores suspension were ultrasonically mixed for 5 min for further analysis.

## 4. Conclusions

In summary, this study developed an FSPCRS-LFB by using flower-shaped PCR scaffolds as capture probes and AuNPs@polyA-cDNA as signal probes, enabling rapid and sensitive detection of *B. cereus* endospores and endospore-cell mixtures in chicken and tea broth. The innovative use of Spacer 18 and PCR produced an aptamer-rich, flower-shaped PCR scaffold, effectively preventing further DNA strand extension. This approach improved the uniformity of RCA scaffolds and stabilized SA-Bio immobilization, increasing sensitivity by 3.7 times compared to SA-Bio, while reducing costs to 87.5% of RCA and 26% of SA-Bio. Additionally, the traditional low-pH method for coupling non-thiolated DNA and AuNPs was enhanced by adding BSA as a blocking agent, improving nanoprobe migration and reducing aggregation.

The FSPCRS-LFB demonstrated rapid detection, delivering results within 15 min. For endospores, the LOD and vLOD were 4.57 and 10^2^ endospores/mL, respectively. For endospore-cell mixtures, the LOD and vLOD were 6.78 and 10^2^ CFU/mL. When applied to chicken and tea broth, the LODs were 74.5 and 52.8 endospores/mL, with recovery rates between 82.19% and 97.88%, demonstrating high sensitivity and reliability across different matrices. The FSPCRS-LFB enabled POCT for *B. cereus* endospores and endospore-cell mixtures, expanding its detection range. The programmability of the flower-shaped PCR scaffold allows for the detection of various targets by simply modifying the aptamer sequence, offering broad applicability and new approaches for foodborne pathogen detection.

## Data Availability

The original contributions presented in the study are included in the article/Supplementary Materials, further inquiries can be directed to the corresponding author.

## Supplementary Materials

The following supporting information can be downloaded at: https://www.mdpi.com/article/10.3390/ijms252011286/s1.
