# Supplementary material for: Flower-Shaped PCR Scaffold-Based Lateral Flow Bioassay for Bacillus cereus Endospores Detection
Source: Int J Mol Sci. 2024 Oct 20;25(20):11286. doi: 10.3390/ijms252011286 (PMC11509332; doi:10.3390/ijms252011286)
Supplement: Supplementary file 1 [file ijms-25-11286-s001.zip › ijms-3249669-supplementary.pdf]

Supplementary Materials

Flower-Shaped PCR Scaffold-Based Lateral Flow Bioassay for *Bacillus cereus* Endospores Detection

Jingjing Tian [1,†], Zhuyi Zhang [1,†], Yaning Shi [1], Zichao Wu [1], Yuting Shao [1], Limin Wang [2], Xinglian Xu [1] and Zhihong Xin [1,*]

[1] Key Laboratory of Food Processing and Quality Control, State Key Lab of Meat Quality Control and Cultured Meat Development, College of Food Science and Technology, Nanjing Agricultural University, Nanjing 210095, China; tianjingjing@njau.edu.cn (J.T.); zyzhang_0615@163.com (Z.Z.); yanings@njau.edu.cn (Y.S.); zichaowu_jason@163.com (Z.W.); 2018208019@njau.edu.cn (Y.S.); xlxu@njau.edu.cn (X.X.)
[2] College of Plant Protection, Nanjing Agricultural University, Nanjing 210095, China; wlm@njau.edu.cn
* Correspondence: xzhfood@njau.edu.cn
† These authors contribute equally to this work.

1. Aptamer Affinity Verification

Synthesize a repeat sequence of the aptamer (Table S1). *B. cereus* cells and endospores were mixed at a 1:4 ratio, centrifuged, and washed twice with 1×BB buffer. The cells were resuspended in 1× BB buffer and stored at 4 °C. Next, 1 nmol of aptamer was denatured at 95 °C for 5 min, cooled on ice for 10 min, mixed with 350 μL of bacterial suspension and incubated at 35 °C, 120 rpm for 30 min. After incubation, the mixture was centrifuged at 8000 rpm, 4 °C for 5 min, and the pellet was washed with 1× BB buffer. The aptamer-bound bacteria were resuspended in 100 μL of 1× TE buffer, heated at 95 °C for 10 min, and cooled on ice for 10 min to release the bound aptamer. After centrifugation at 12,000 rpm for 5 min, the supernatant containing the released aptamer was collected as a template for PCR validation. A 50 μL PCR system was prepared with templates, 0.025 $U \cdot \mu L^{-1}$ rTaq DNA polymerase, 0.4 μM each of forward and reverse primers, 250 μM dNTPs, and 1× PCR buffer (Mg^{2+} plus). The PCR program included 98 °C for 10 min, followed by 30 cycles of 98 °C for 60 s, 57 °C for 60 s, and 72 °C for 60 s, with a final extension at 72 °C for 10 min.

2. Endospore Extraction

Centrifuge an appropriate amount of *B. cereus* culture (over 48 h) at 4 °C, 8000 rpm for 5 min. After removing the supernatant, the cells were resuspended in pre-chilled ultrapure water and shaken at 170 rpm for 60 min at 4 °C to break the cells and release endospores. The mixture was centrifuged at 4 °C, 15,000× *g* for 60 min, resulting in two distinct layers: endospores at the bottom and cell debris above. The supernatant was carefully removed, preserving the endospores. Ultrapure water was added gently along the tube wall to resuspend the cell debris while protecting the endospore layer. After mixing at 1000 rpm, the supernatant was removed again. This process was repeated if necessary, and the purified spores were stored in pre-chilled ultrapure water at 4 °C.

3. Collection and Storage of Endospore-Cell Mixture

The endospore-cell mixture consists of *B. cereus* in logarithmic and stationary phases. Log-phase cells were obtained by adding 60 μL of culture to 30 mL LB medium and incubating at 35 °C until OD_{600} = 1. Stationary-phase cells were obtained by incubating 60 μL of culture in 30 mL LB medium at 35 °C for 48 h. The two phases were mixed at a 1:4 ratio, centrifuged at 4 °C, 10,000 rpm for 5 min, and washed twice with 1× BB buffer. The final cell suspension was prepared in 600 μL BB buffer and stored at 4 °C for future use.

4. The Calculation Method of *B. cereus* Cell Count

A single colony was cultured at 35 °C until OD_{600}=1. Then, 100 μL of the culture was diluted in 900 μL of sterile 9% saline for serial dilutions, yielding bacterial suspensions with dilutions from 10^1 to 10^8. Plates were spread with dilutions from 10^3 to 10^7 and incubated at 35 °C for 24 h. Colonies between 30–300 were counted. For *B. cereus* endospore counting, a similar method was used: the culture was incubated at 35 °C for 48 h, heated at 80 °C for 15 min, and then serially diluted and plated. The number of cells in 1 mL of culture was calculated using the formula: Number of cells in 1 mL = Average colony count × Dilution × 10.

Table S1. Sequences of oligonucleotides used for aptamer-target affinity verification.

Name	Sequence (5′-3′)
Aptamer	TCGTGCCGGTTGGTAGGATCtttATGGGCTACTGGAGCATCTGttt<br>ATGGGCTACTGGAGCATCTGtttATGGGCTACTGGAGCATCTGttt<br>GAGCATGACGCACTGTCAGG
Primer-F	TCGTGCCGGTTGGTAGGATC
Primer-R	GAGCATGACGCACTGTCAGG

Table S2. Sequences of oligonucleotides used for flower-shaped PCR scaffold-based lateral flow platform.

Name	Sequence (5′-3′)
DNA$_T$-F	ATGGGCTACTGGAGCATCTGTTTTTTTspacer18TTAGTATATGGCACAATCTGAC
DNA$_T$-R	ATGGGCTACTGGAGCATCTGTTTTTTTspacer18TTAAGTTTCTTCACGAAGTCC
DNA$_C$-F	*CACTCTATAATAATAAT* TTspacer18TT AGTATATGGCACAATCTGAC
DNA$_C$-R	*CACTCTATAATAATAAT* TTspacer18TT TAAGTTTCTTCACGAAGTCC
polyA-cDNA	AAAAAAAAAAAAAAA TTAT *ATTATTATTATAGAGTG* AAAAA CAGATGCT

Note: Sequences with underlining or bold italics indicate complementary sequences.
